# Lung Ultrasound Artifacts Interpreted as Pathology Footprints

**DOI:** 10.3390/diagnostics13061139

**Published:** 2023-03-16

**Authors:** Marcello Demi, Gino Soldati, Alessandro Ramalli

**Affiliations:** 1 Department of Bioengineering, Fondazione Toscana Gabriele Monasterio, 56126 Pisa, Italy; 2 Ippocrate Medical Center, 55032 Lucca, Italy; 3 Department of Information Engineering, University of Florence, 50139 Florence, Italy

**Keywords:** lung ultrasound, b-lines, vertical artifacts, pulmonary artifacts, physical models

## Abstract

Background: The original observation that lung ultrasound provides information regarding the physical state of the organ, rather than the anatomical details related to the disease, has reinforced the idea that the observed acoustic signs represent artifacts. However, the definition of artifact does not appear adequate since pulmonary ultrasound signs have shown valuable diagnostic accuracy, which has been usefully exploited by physicians in numerous pathologies. Method: A specific method has been used over the years to analyze lung ultrasound data and to convert artefactual information into anatomical information. Results: A physical explanation of the genesis of the acoustic signs is provided, and the relationship between their visual characteristics and the surface histopathology of the lung is illustrated. Two important sources of potential signal alteration are also highlighted. Conclusions: The acoustic signs are generated by acoustic traps that progressively release previously trapped energy. Consequently, the acoustic signs highlight the presence of acoustic traps and quantitatively describe their distribution on the lung surface; they are not artifacts, but pathology footprints and anatomical information. Moreover, the impact of the dynamic focusing algorithms and the impact of different probes on the visual aspect of the acoustic signs should not be neglected.

## 1. Introduction

More than thirty years ago, Lichtenstein and colleagues described lung ultrasound (US) signs of interstitial lung disease in terms of artifacts [1]. Since then, terms such as A-lines and B-lines have been consistently present in the literature related to the US exploration of the lung. A-lines and B-lines are usually associated with normality and with a sign of interstitial pathology, respectively [2,3,4,5,6]. Over the years, this pathology has included extravascular water of the lung, diffuse interstitial diseases of the lung, contusions and bleeding, and inflammatory states [7], that is, anything that alters the relationship between air and lung tissue without consolidating the organ [8]. The original observation that the US exploration of the still aerated lung informs us about the physical state of the organ rather than the morphological (anatomical) details related to the disease has reinforced the idea that the B-lines represented US artifacts [9]. Obviously, the B-lines do not represent any anatomical part of a pathological lung as, similarly, the A-lines do not represent the anatomy of a normal lung. However, the definition of artifact does not appear adequate in our case. According to the Cambridge dictionary, “an artefact is something observed in a scientific investigation or experiment that is not naturally present but occurs as a result of the preparative or investigative procedure”. According to the Merriam-Webster dictionary, “an artifact can be a defect in an image (such as a digital photograph) that appears as a result of the technology and methods used to create and process the image or it can be a product of artificial character (as in a scientific test) due usually to extraneous (such as human) agency”. According to Collins, “an artifact is any non-natural feature or structure accidentally introduced into something being observed or studied”. All these definitions lead us to consider the existence of something false, of something wrong, or something conditioned by the process that produces the artifact.

On the contrary, pulmonary US signs have over the years shown considerable diagnostic accuracy, which has been usefully exploited by physicians in numerous pathologies. Although these signs do not explicitly reproduce anatomical details of the lung, they represent practical clinical information. Moreover, recent results provided by numerical simulations and experimental results obtained in the laboratory on physical lung phantoms have increased the robustness of the clinical information contained in the US lung signs [10,11,12,13,14,15]. Thanks to these results, what was before considered artefactual information has been decoded as physical information of the lung surface. The primary purpose of this paper is to outline how the so-called vertical artifacts are the responses of acoustic traps located on the lung surface and, consequently, to raise these acoustic signs to the level of anatomical information [13,14]. Secondarily, the paper aims to recall the main characteristics of the trap acoustic signs, describe the method that has been used to comprehend their genesis, and highlight their relationship with the surface histopathology of the lung. Moreover, the impact of the US equipment on the visual characteristics of the trap acoustic signs is addressed, and two important sources of potential signal alteration, which remain to be investigated, are illustrated.

## 2. Materials and Methods

**Figure 1:**
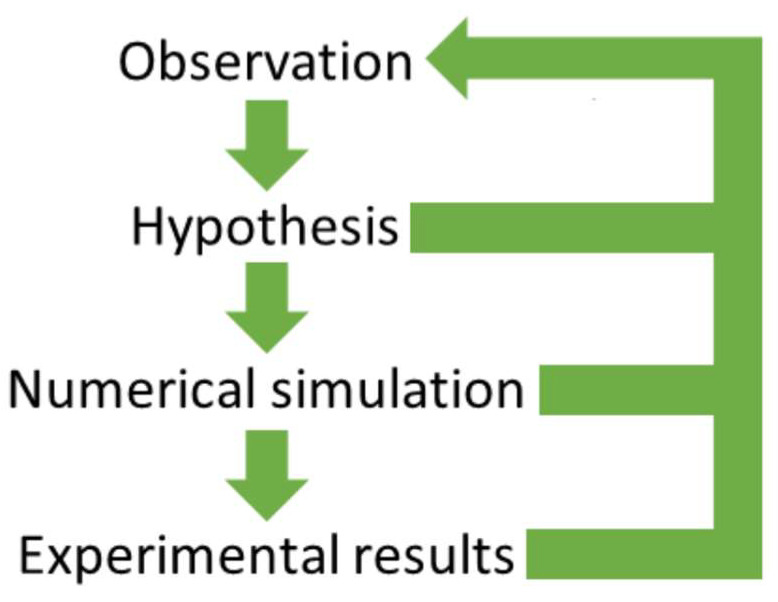
Schematic representation of the methodological approach used to investigate the genesis of the US signs, which are observed on lung US images in the presence of pulmonary pathologies.

Our interest in lung US as a team began in 2010, and just after a few months, we adopted a precise method of analysis which, in the subsequent years, provided significant results: the four-step method [13,14,16], which is illustrated in Figure 1.

The first step is to watch video clips of lung US exams, looking for ideas on the genesis of the observed acoustic signs. The second step is to formulate a reasonable hypothesis derived from, and consequently congruent with, the physics of acoustic wave propagation. The third step is to look for confirmation of the hypotheses by simulating the imaging process on numerical phantoms. The fourth and last step is to verify the hypotheses through experimental results on calibrated physical phantoms. As can be seen in Figure 1, a retroactive role of the previous steps is planned for each single step. For example, once a hypothesis that is based on the observation of numerous video clips is formulated, it is important to conduct a second round of visual analysis of the same video clips. This is because human vision is not limited to simple retinal vision. Important tasks of our vision system are explicated by the brain, and once a hypothesis is formulated, the brain setting changes, and new useful details can be captured on the same video clips.

The visual characteristics of the acoustic signs depend on many imaging parameters [14,17]. However, two important sources of signal alteration, which have never been considered before, have been highlighted during our analysis. Hereinafter, if not differently specified, images were acquired through the US advanced open platform (ULA-OP) [18]. This is a hardware-based research scanner [19] that allows the high programmability and flexibility needed to control and set specific imaging parameters, both in transmission and reception.

## 3. Results

### 3.1. The Four-Step Method

At first, we observed that the acoustic signs were oriented along the specific scan/imaging direction [20]. This observation led us to formulate the hypothesis of the acoustic trap [10] as a logical explanation of the acoustic signs observed on lung images: the pulse energy is partially “trapped and repeatedly bounced” in a spot on the lung surface, i.e., the trap, and is subsequently released while the probe and the system are still receiving and processing the RF signal from that direction.

A question, however, was raised: what mechanism, which is congruent with pulmonary anatomy, can trap the pulse energy? Two plausible hypotheses were analyzed: air bubble vibrations and multiple reflections between air bubbles. Both theoretical and experimental results provided the answer. From the Minnaert relationship [21,22], we know that an air bubble (for example, a partially filled alveolar sac) can vibrate once it is excited by an acoustic wave, but cannot re-radiate an US wave at radio frequency (2–6 MHz) unless the bubble is a microbubble with a diameter of a few microns. Experimental results also confirmed that isolated air bubbles do not give rise to acoustic signs [10]. Conversely, numerical simulation and experimental results showed that tissue mimicking volume surrounded by air bubbles (both tissue and bubbles, having sizes compatible with the pulmonary anatomy) can easily give rise to acoustic signs in the range of 2–6 MHz. This second observation suggested the plausible hypothesis that the observed acoustic signs are generated by multiple reflections of an acoustic wave propagating in a non-aerated medium (the interstitial medium) surrounded by aerated structures (the alveoli sacs) [10].

**Figure 2:**
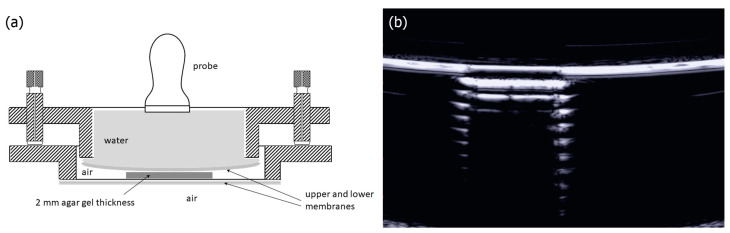
(**a**) Shows an agar disk positioned between two polyethylene films with the upper film that rests only on the central part of the agar disk. (**b**) Shows how the agar disk generates a short reverberation in the center and longer reverberations on the periphery where the agar is partially limited by air even at its upper wall.

The third observation was related to the length of the trap acoustic signs. Physicians consider those signs that start at the pleura plane and extend to the bottom of the screen to be important [1,5]. Today, we know that this is not true and that the extension of the acoustic signs strongly depends on the imaging parameters [14,17], which often do not extend to the bottom of the screen, and that even those acoustic signs are extremely interesting for clinicians. However, even though they do not extend to the bottom of the screen, their length is an important feature, according to the clinicians’ experience. A 12 cm long US sign was obtained for the first time in the laboratory when an agar gel volume completely surrounded by air, except for a 1 mm aperture at the top, was used [14]. However, the illuminating result was casually obtained later when a 2 mm agar disk was used as a phantom. The agar disk was positioned between two polyethylene films; the upper film was covered by a bed of water, which guaranteed a good acoustic matching between the agar disk and the probe, and the lower film, where the agar disk was sitting, separated the latter from the underlying air. The upper film under the weight of the water assumes a convex shape and rests only on the central part of the agar disk (Figure 2a). Figure 2b shows how the agar disk gives rise to a short reverberation in the center and longer reverberations on the periphery, where the agar is partially limited by air, even at its upper wall. Since the beam size is not infinitesimal, once it is reflected by the air underlying the lower film, it is partially intercepted by the air that separates the periphery of the disk from the water laying on the upper film. Consequently, the number of multiple reflections within the agar disk increases and, as a consequence, the length of the acoustic signs also increases. This casual experimental result clearly shows how the length of the trap acoustic sign (i.e., the time interval during which the trapped energy is gradually re-radiated) depends on the shape of the acoustic trap.

**Figure 3:**
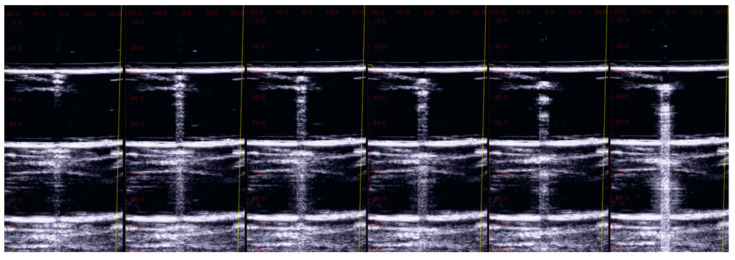
Example frames from a video clip during which a drop of water was acquired, with a linear US probe, while it was falling from a water container. From left to right, the number of observed reverberations and their spacing increase. The last frame shows how the trap acoustic sign changes radically (it appears brighter and much longer) when the drop reaches the necessary weight to detach and fall.

In order to further verify the previous hypothesis, a video clip was acquired on a singular phantom, i.e., a drop of water falling from a water container. Initially, the drop is a hemisphere of water attached to the water container by means of a large surface. However, as its weight increases, it assumes the characteristic shape of a drop of water attached to a surface for a peduncle. Such a shape completely changes the response of the drop to the US since it is now limited by air even at the top, apart from a small channel of water that keeps the drop attached to the container. Now, two high acoustic impedance discontinuities (both below and above) reflect the acoustic waves, and the internal reverberation has a longer duration. Let us make a small hole in a polyethylene film that retains a bed of water, and let us acquire a US video clip of a drop of water while it is falling. Figure 3 shows some frames of the video clip. Initially, a short reverberation generated between the bottom of the drop and the polyethylene film is observed. Subsequently, as the size of the drop increases, the distance between the horizontal lines provided by the reverberations increases. The number of visible reverberations also increases until the drop reaches the necessary weight to detach and fall. In these last frames, the acoustic sign changes radically: it appears brighter and much longer. The length of the acoustic sign does not depend only on the volume of the drop, but it also depends on the liquid channel that connects the drop to the polyethylene film. The acoustic trap should be seen as an assembly of two elements: a channel that links the trap to the external medium and the chamber of the trap. The two examples given by the agar disk and by the drop of water show the importance of the experimental results obtained on physical phantoms (the fourth step).

**Figure 4:**
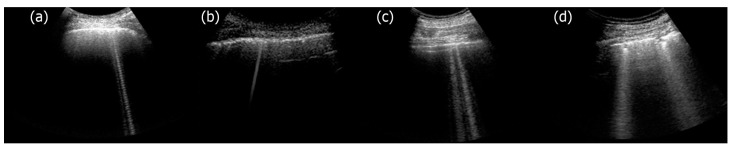
Lung US images acquired with a Toshiba Aplio XV scanner and a PVT-375BT convex probe are shown. From left to right, (**a**) a modulated acoustic sign, (**b**) an acoustic sign with a uniform gray level, (**c**) slight confused acoustic signs, and (**d**) confused acoustic signs starting beyond the pleura line are shown, respectively.

The importance of the third step was highlighted when another observation was analyzed. Physicians distinguish acoustic signs from modulated acoustic signs [23], and great importance is given to this distinction. Mostly, trap acoustic signs have a confused structure, but sometimes they have a clear periodical structure given by a cascade of small horizontal bright segments; these are the signs that physicians call modulated artifacts (Figure 4a). The genesis of these signs has been explained through the modulation transfer function of the acoustic trap (i.e., through its spectral signature). Due to the internal multiple reflections of the acoustic pulse and due to their constructive and destructive summations, the power spectrum of the acoustic signal, which is re-radiated from the trap, is given by a set of regularly spaced harmonics of the pulse spectrum, where the frequency intervals are inversely proportional to the sizes of the trap. For example, if the shape of an acoustic trap could be approximately defined by three dimensions d_1_, d_2_, and d_3_, then its spectral signature would be characterized by the harmonics f_n,m,l_:

(1) $$f_{n,m,l}=\frac{c_{0}}{2}\sqrt{{\left(\frac{n}{d_{1}}\right)}^{2}+{\left(\frac{m}{d_{2}}\right)}^{2}+{\left(\frac{l}{d_{3}}\right)}^{2}}$$

where n, m, and l are integer numbers, and c_0_ is the US propagation speed [24]. Equation (1) explains the different modulations that are observed in the lung US signs, which can also be replicated with a simple numerical simulation. The numerical simulation, however, provided an unexpected result. If the three dimensions d_1_, d_2_, and d_3_ were reduced to a single dimension d (for example, if the trap shape was approximately spherical) and d was small, then the set of harmonics f_n,m,l_ could be reduced to a single harmonic (in the frequency range of the probe). If a single harmonic was thus obtained, the image formation process would generate an acoustic sign with a uniform gray level. Such an acoustic sign had never been observed until then, and that challenged our hypothesis. A return to the first step (observation) was mandatory, and while watching the same video clips again, we surprisingly found the acoustic signs we had not previously noticed (Figure 4b). This anecdote illustrates the importance of numerical simulation, which should not be considered a simple verification process. Numerical simulation can provide important and unexpected suggestions that can further validate or reject the hypotheses under examination.

**Figure 5:**
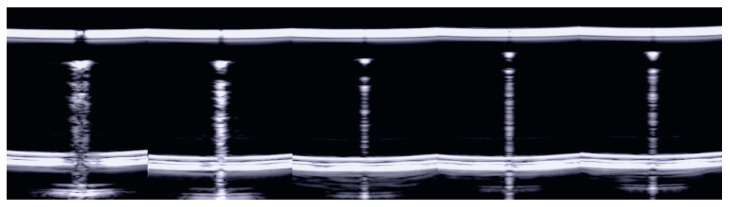
From right to left, the US images show how a modulated acoustic sign generated by a physical phantom becomes confused when the size of the trap input channel gradually increases.

Some acoustic signs, however, are confused (Figure 4c). Often, they do not have the periodical structure of the modulated signs, and this is the fifth observation we analyzed. We do not have a definitive physical explanation for the latter yet. Experimental results on isolated traps and theoretical speculations suggest an explanation that, however, must be further explored. In a previous paper [13], it was shown how a modulated acoustic sign generated by a physical phantom becomes confused when the size of the trap input channel gradually increases (Figure 5). Here, it is suggested that the long modulated acoustic signs are probably generated by isolated acoustic traps that are connected to the thoracic wall through small interstitial channels. In this case, the trap acts as a point-like source of US and, consequently, can both re-radiate the trapped energy slowly and eliminate the uneven acoustic perturbation of the particles of the medium at the top of the channel.

This conclusion, based on theoretical speculations and experimental results, partially contradicts the theory of the spectral signature, according to which the modulated structure of the acoustic signs depends on the size of the traps. Here, we see that the structure of the trap acoustic sign depends also on the size and shape of the input channel. The response of an isolated acoustic trap to an US pulse should be analyzed as a function of both the acoustic channel and the trap chamber. The similitude with the Helmholtz resonator [25] is evident, even though the frequency range is completely different.

A more complex circumstance arises when non-isolated traps or traps having multiple input channels are considered. So far, these traps have not been specifically analyzed, even though a partial explanation has been given as an appendix of the White Lung investigation [10]. The so-called White Lung artifact was investigated through numerical simulation. The hypothesis that was formulated, through the visual analysis of numerous video clips acquired on different patients and the examination of their clinical diagnoses, was that of a distribution of small, aerated spaces, separated by thickened interstitial spaces. A stochastic process where every single reflection inside a distribution of traps was characterized by its own delay and amplitude was assumed. In addition to suggesting a physical explanation of White Lung, the same process also suggests a physical explanation of the local acoustic signs with a confused structure, which are observed, for example, in patients with pulmonary fibrosis or ARDS. The mathematical model provided images and acoustic signs that were very similar to those provided by the clinical cases of White Lung and fibrosis, respectively. Moreover, this is in line with what has been observed both in patients and on phantoms. In patients [26], the lung US images show how the US pattern changes by increasing the alveoli internal pressure and, consequently, their size and dominance with respect to the surrounding lung tissue. Analogously, on phantoms [27], US images show how the US pattern changes by increasing the dominance of the air spaces with respect to water. These articles show how the US pattern changes from White Lung to multiple unstructured acoustic signs and, subsequently, to isolated modulated signs by progressively increasing the dominance of the air spaces.

### 3.2. The Impact of Wrong Utilization of the Ultrasound Equipment

Every commercial US scanner uses a dynamic focusing algorithm in receive mode [28,29,30,31] to increase the quality of the acquired images. Once an US pulse is sent in a direction *d_r_*, a scatterer *s_c_*, located along this direction at a distance *d* from the probe, partially reflects the acoustic wave and generates an echo signal *s*(*t*). Therefore, the *N* receiving elements, lined up on the head of the probe, receive *N* signals *s*(*t*–*t_i_*), where *t_i_* is the time shift caused by the geometry of the receiving system. Consequently, in order to focalize the response of the scatterer *s_c_*, the signals *s*(*t*–*t_i_*) are added by compensating the time shift *t_i_*. However, since the time shift *t_i_* depends on the distance *d* of the scatterer *s_c_* from the probe, then the time shift *t_i_* changes for every scatterer located along the direction *d_r_*. The idea is to correctly focalize the response of every scatterer located along the scan direction *d_r_*, even if their distance from the probe varies. Consequently, a dynamic focusing algorithm is used in receive mode to vary the values of the time shift *t_i_* as a function of the time *t*. The dynamic focusing algorithm (and the dynamic apodization algorithm, too) in receive mode increases the quality of the images when echoes coming from scatterers at different distances from the probe are expected. On the other hand, when a signal that has been radiated by an acoustic trap located at a fixed distance from the probe has to be analyzed, these algorithms should not be used since they introduce artifacts. In this case, the term artifact is correctly used since the two algorithms (the dynamic focusing and the dynamic apodization algorithms) alter the true signal. According to Collins, they accidentally introduce non-natural features into something being observed or studied.

**Figure 6:**
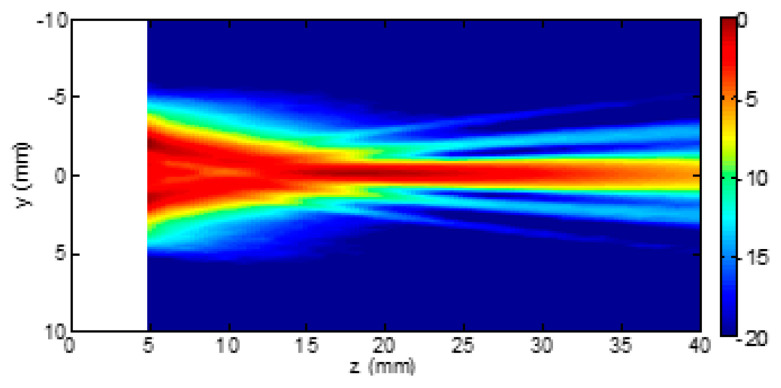
Elevation beam profile of the Esaote LA533 linear array probe when pulses with a central frequency of 4 MHz are used. Figure courtesy of Esaote, Florence, Italy.

**Figure 7:**
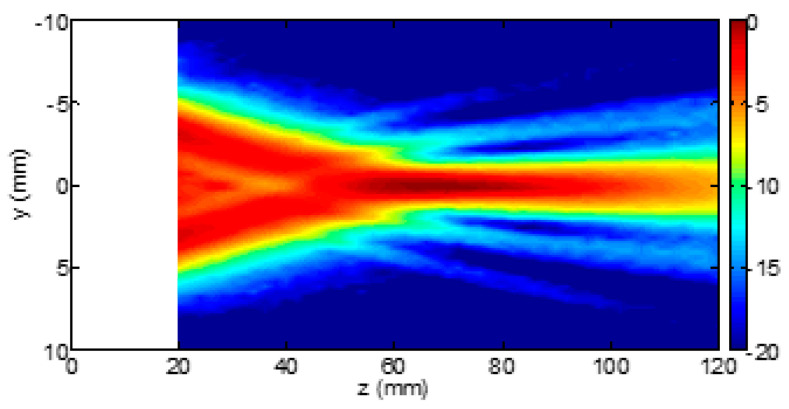
Elevation beam profile of the Esaote CA631 convex array probe when pulses with a central frequency of 4 MHz are used. Figure courtesy of Esaote, Florence, Italy.

Usually, when the process of beam formation is analyzed, we refer to lateral focusing. Nevertheless, another source of data alteration is introduced by the elevation focus [29]. The latter is typically static (unless probes with active elements arranged in a matrix are used) since it is defined by the probe acoustic lens. This lens is designed to fix the elevation focus at the supposed center of the region of interest, which depends on the application. The problem is not trivial since, in lung US, clinicians use linear, convex, and cardiac probes indifferently. While the linear probe has been designed to study superficial structures, and, consequently, the focus of the acoustic lens is close to the head of the probe, the convex and the cardiac probes have been designed to study deeper structures, and the focus of their acoustic lens is far from the head of the probe. For example, Figure 6 and Figure 7 show the beam profiles in elevation of the LA533 linear array probe from Esaote and of the CA631 convex array probe from Esaote, respectively, when pulses with a central frequency of 4 MHz are used.

Let the distance between the head of the probe and the pleura plane be approximately 20 mm. Then, the difference between the two beam sizes at the pleura plane is evident and substantial. The beam size is 2 mm or 10 mm when the LA533 or the CA631 is used, respectively. The LA533 is correctly used since its acoustic lens focalizes the beam at a mean distance of 20 mm, i.e., the LA533 has been designed and tuned to analyze structures at a mean depth of 20 mm. Convex and cardiac probes are not used correctly when they are used to analyze the pleural plane since they have been designed and tuned to analyze deeper structures. An increased size of the beam decreases the spatial resolution of the probe, and an irregular distribution of the beam energy (as in the case of the CA631 at the depth of 20 mm) alters the response of the pleural plane yielding to artifacts.

## 4. Discussion

After birth, the surface of the lung shows a porous structure, and in many pathologies, which still allow the aeration of the pulmonary cortex (the so-called interstitial pathology), the lung maintains a superficial porosity, with variable degrees of density and morphology depending on the pathology [32,33]. This also occurs when the lung tissue partially collapses, and the tissue does not show pathological changes. In these contexts, the assumption that the tissue component of the pleural surface may give rise to a distribution of acoustic channels and traps, surrounded by air spaces, is reasonable. In this case, the insonation of the pleural plane locally allows the trapping of the acoustic energy and its gradual re-radiation to the probe. These systems of channels and traps are normally present along the pleura (the normal interstitium), but the small size of the channels (often no more than 10 microns [34]) does not allow the transmission of a significant quantity of acoustic energy to the underlying tissue component. It is only when these channels widen, due to the presence of an interstitial pathology, that they allow the transmission of a quantity of energy that is sufficient to generate a visible acoustic sign during the re-radiation of the trapped energy [35].

A subpleural pathology of the lung, which allows the organ to maintain a certain degree of dispersed aeration, generates acoustic signs, as clinical evidence has repeatedly confirmed [6,36,37]. Similarly, deflation of the lung can produce focal densities in relation to the selective collapse of subpleural peripheral air spaces (folding of air spaces), with a critical reduction of aerated units [38]. Since the distribution of the acoustic traps represents the superficial (pathological or pathologically deflated) interstitium of the lung, a sort of parallelism between the distribution of the trap acoustic signs and the superficial histopathology of the lung sounds realistic.

While the concept of non-consolidated density has already been expressed in previous papers [8,39], no clear clinical interpretation has yet been made regarding the distribution and the visual characteristics of the trap acoustic signs (and, consequently, of the focal thickening of the pulmonary superficial, non-aerated medium). What is known is that different interstitial diseases of the lung may or may not involve the surface, and, consequently, that their US visibility depends on the surface involvement. Moreover, some of these diseases appear to be more evident in specific regions of the lung. Some diseases largely affect the surface of the lungs, while others show a preference for a location or a patchy appearance. These clinical aspects have already been described when focusing on the differential diagnosis between primitive pulmonary processes (fibrogenic diseases) and cardiogenic diseases [40,41]. Recent experiences with pneumonia from COVID-19 have been enlightening, and the distribution of the acoustic traps in these contexts offers a coherent explanation [23]. The acoustic trap theory allows clinicians to estimate the gravity of a specific pathology through the analysis of the distribution of the acoustic signs. Physiological acoustic traps of the normal lung cannot generate visible acoustic signs. Consequently, the more numerous the acoustic signs, the more severe the pathological involvement is. However, apart from the density and the topographic distribution of the trap acoustic signs, apart from their continuous or patched distribution, and apart from the appearance or not of White Lung, the acoustic trap theory suggests that the visual structure of the acoustic signs may provide information regarding the geometry of the trapping systems. In other words, an acoustic sign can be considered a pathology footprint. The trapping system, with its combinations of acoustic channels and traps with variable characteristics, can roughly indicate groups of defined histopathologies.

The acoustic interaction of US with the surface of a lung when the latter is affected by an interstitial disease involves many traps with variable characteristics, such as size, morphology, and content. According to this, some correlations can be speculated. For example, a regular distribution of acoustic traps with small openings (channels) on the pleural surface is likely to generate images with many extended acoustic signs, starting from the pleura and having a modulated structure. Conversely, large access channels to acoustic traps would tend to produce larger, non-modulated, and often non-extended signs. This clear difference is observed when subjects with initial pulmonary edema (small channels derived from interlobular septa) and subjects with idiopathic pulmonary fibrosis are evaluated (large channels with variable size due to collagenization) [40]. Another aspect that must be considered is the visual localization of the bottom of the acoustic trap. The acoustic signs originate from the bottom of the trap, and, consequently, the depth of the trap can be estimated when they do not appear to originate from the pleural line (Figure 4d). Therefore, a trap acoustic sign that appears as starting beyond the reflection of the pleural line can indicate a primitively pulmonary (even micronodular) genesis because the initial hydrostatic edema (cardiogenic) does not produce acoustic traps with these characteristics [42].

The correlation between surface histopathology and the visual characteristics, distribution, and gray-level intensity of the phenomena currently defined as vertical artifacts will probably strengthen soon. The specificity of the acoustic signs will probably point clinicians toward specific groups of pathologies. In this sense, the epidemic of pneumonia from COVID-19 has taught us a lot [23,43], especially thanks to the variety and the temporal evolution of the finds. In COVID-19, early alveolar aspects appear as discontinuous acoustic signs associated with discontinuous areas of White Lung. Discontinuous acoustic signs are probably generated by discrete distributions of small channels that transmit, trap, and gradually re-radiate the acoustic energy. As for White Lung, its origin from the insonation of groups of homogeneous and closely packed small traps is conceivable. The presence of wide non-modulated signs, originating from the bottom of identifiable larger traps (the micro-consolidations), is only observed with the consolidating evolution of the disease. It is interesting to note that this evolution and the heterogeneity of findings is constant in all situations of alveolar damage (ARDS is the most typical case).

## 5. Limitations

One limitation of the method that has been introduced and illustrated in Section 2 is the difficulty to produce small durable phantoms. Calibrated physical phantoms with reproduced aerated volumes and tissue-mimicking septa that have dimensions similar to the real lung are needed to further investigate the US signs, but realizing the latter is challenging. Moreover, durable phantoms are also needed to guarantee the repeatability of the experimental step (the fourth step of the method). The agar gel that has been used so far is not suitable since, once exposed to air, it begins to dry, and its acoustic properties change. More complex mathematical models must be developed, and new materials must be explored to realize the physical phantoms [44,45].

The impact of the imaging parameters is another limitation of the methodology [14,17]. For example, it is difficult to distinguish the contribution of the imaging parameters to the length of a trap acoustic sign from the contribution of the trap geometry. Specific acquisition protocols could even be developed where every imaging parameter is exactly defined. However, differences between different US scanners and differences between different probes cannot be avoided.

## 6. Conclusions

Over the years, different terms have been used to highlight the lung US acoustic signs as opposed to the anatomical information. The terms ring-down artifact, comet-tail artifact, and vertical artifact have also been proposed, in addition to B-line. The two terms ring-down artifact and comet-tail artifact have been proposed in different papers by different authors and derive from analogies with similar phenomena suggested by the visual aspect of the examined acoustic sign. The comet-tail artifact [46] was described as a reverberation effect, and the ring-down artifact [47] was described as a resonance effect. All these terms have a common attribute: they consider trap acoustic signs as artifacts. However, all of them refer to trapped (absorbed, transmitted, etc.) energy that is subsequently released in the form of a periodic or confused signal. The acoustic traps can be seen as secondary sources of US. Consequently, trap acoustic signs highlight the presence of acoustic traps and quantitatively describe their distribution on the lung surface; they are not artifacts, but pathology footprints and anatomical information.

The term artifact does not do justice to the trap acoustic signs that are observed on US images of the lung. These acoustic signs are reproducible in the laboratory [13] and convey important clinical information. Even though they do not represent anatomical details of the lung in the proper sense, they originate from altered spots (acoustic traps) of the lung surface and represent a structural reality of the pathological lung. 

It is undeniable that the diagnostic usefulness of lung US is limited to the lung pathologies that surface on the pleural plane. Lung US cannot provide information on deeper layers of the lung unless its aerated component is strongly reduced. However, the methodology that has been adopted to investigate the genesis of the acoustic signs has provided important results. Physical explanations of the main attributes of trap acoustic signs, such as their length, brightness, and shape, have been formulated thanks to the four-step method. Different approaches to the study of the information provided by lung US, such as those derived from artificial intelligence, could not have helped us to develop the physical knowledge of lung US that we have today. This research method is somehow comparable to the methodology that usually guides the clinical use of thoracic US (a practice that is known as Clinical US [48]).

The visibility and the visual characteristics of the acoustic signs, which are potentially generated by the traps once they have been activated by an US pulse, depend both on the trap geometry and the imaging parameters. The impact of the latter on the acoustic signs generated by specific traps is generally known, and, in our opinion, the problem can be partially overcome by analyzing how the response of the acoustic traps varies when varying the imaging parameters. However, two important sources of signal alteration are still ignored: when exploring the pleural plane of the lung, the dynamic focusing and apodization should be switched off. Moreover, given the geometric features of the acoustic lens, the impact of different probes on the visual aspect of trap acoustic signs should not be neglected.

Much work has still to be done, but in our opinion, the diagnostic potential of lung acoustic signs will progressively increase through a better understanding of the relationship between the visual aspects of these signs and the pathological morphology of the lung surface. What has so far been interpreted as an artifact should be perceived as a pathology footprint.

## Data Availability

Not applicable.
